# Phylogenetic relationships and genetic differentiation of two *Salamandrella* species as revealed via COI gene from Northeastern China

**DOI:** 10.1371/journal.pone.0298221

**Published:** 2024-02-14

**Authors:** Wanli Liu, Zhuo Duan, Dingcheng Wang, Wenge Zhao, Peng Liu

**Affiliations:** 1 College of Life Science and Technology, Harbin Normal University, Harbin, Heilongjiang Province, China; 2 Key Laboratory of Biodiversity of Aquatic Organisms, Harbin Normal University, Harbin, Heilongjiang Province, China; National Cheng Kung University, TAIWAN

## Abstract

Due to traditional classification methods’ limitations, some cryptic species remain undiscovered. To better explore the existence of the Schrenck salamander (*Salamandrella tridactyla*, a cryptic species of Siberian salamander *S*. *keyserlingii*) in China, we conducted a molecular phylogenetic analysis to confirm the taxonomic relationship among *Salamandrella* species and investigate genetic variation. We used complete sequences of the mitochondrial COI gene from 65 specimens collected across a wide range in Northeastern China. Thirty-five haplotypes were obtained from six populations. They showed medium-high haplotype diversity (H_d_) and low nucleotide polymorphism (π). The phylogenetic tree and haplotype network analysis revealed that populations from Greater Khingan Ridge (Huma: HM) and Lesser Khingan Ridge (Tieli: TL) belong to *S*. *keyserlingii*, while populations from Changbai Mountain (Shangzhi-zhuziying: SZ, Shangzhi-cuijia: SC, Hailin: HL, and Baishan: BS) belong to *S*. *tridactyla*. This indicates the monophyly of *Salamandrella* and each of the two species. There was a substantial level of genetic differentiation between different species and within populations of the same species. This differentiation was significantly related to geographical distance. At last, the mismatch distribution and neutrality analyses indicated that the TL populations have undergone expansion of history. The study supplements the distributional range of Schrenck salamander. And it provides a theoretical basis for species conservation of *Salamandrella* species.

## Introduction

The most northerly and widely distributed genus of the Hynobiidae, *Salamandrella* is found in Siberia, Northeast China and Inner Mongolia, and Hokkaido, Japan. There are certain differences in the appearance of different regions. There are currently two species of this genus. The Siberian salamander (*Salamandrella keyserlingii*, Dybowski, 1870) displays a North Palaearctic distribution pattern. It boasts the broadest and northernmost geographic range among the over 500 recognized, extant urodelan amphibians. From Hokkaido in the East to the Northeast of Europe in the West, and from the Chukotkan peninsula in the North to Northeast China in the South, this species extends over a vast territory [1–3]. *S*. *keyserlingii* is LC (Least Concern) on the IUCN Global Red list and under State protection category II in China. Molecular analyses of both mitochondrial and nuclear genomic data have revealed the existence of a cryptic species, the Schrenck salamander (*S*. *tridactyla*, Nikolsky, 1905), in the southeast of Russia (Primorye and southern Khabarovsk krai) and northeast of China [1, 2, 4–9]. *S*. *tridactyla* is listed as NE (Not Evaluated) on the IUCN Global Red list. However, due to previous encroachment on their habitats, the future of these "living fossil" species is uncertain, and understanding their survival status has become urgent. The exact distribution of the species, particularly the northern boundary of *S*. *tridactyla*, which is the zone of contact between the two *Salamandrella* species, has not been well delimited. Therefore, it is now necessary to reevaluate the taxonomic status and outline genetic affinities of *Salamandrella* populations from areas adjacent to the known range of *S*. *tridactyla* [1]. In this connection, regions of Northeastern China, that are probably key in this regard remain poorly studied [10, 11].

Northeastern China is hypothesized to be the center for the origin of Hynobiids. There are 3 living genera (*Salamander*, *Hynobius* and *Onychodactylus*) and 3 fossil genera (*Chunerpeton*, *Liaoxitriton*, and *Jeholotriton*) in this region [12]. It has been confirmed that *Salamander* exhibits high levels of mtDNA divergence in the Amur River region and Northeastern China. The *Salamandrella* population (i.e. *S*. *tridactyla*) in the Primorye region is closely situated to Northeastern China. Both *S*. *keyserlingii* and *S*. *tridactyla* are widely distributed and easily identified in the aforementioned regions [2, 9, 11, 13, 14]. However, molecular genetic methods are necessary to distinguish between individual *Salamandrella* species. Because they are morphologically identical with only minor biological differences (Individuals in various geographic populations had a total length of 109.03 ± 1.46 mm and a body length of 49.79 ± 0.82 mm) [2, 4, 15]. As is well known, DNA barcoding marker and tree-based methods can provide a convenient, accurate and valid tool to identify species and delimit boundaries. So it has been widely used in phylogenetic surveys among Asiatic salamanders populations and/or closely related species [12, 16–20].

Previous studies utilizing mtDNA gene haplotypes to construct phylogenetic trees have revealed that *Salamandrella* species in Northeastern China can be divided into two distinct clusters. Samples (HM037736, HM037761, HM037786, JX508746, JX508747, DQ333814, XM2055, XM2056, XM2057, XM2059, XM1879, XM1880, GQ849165, GQ849165) collected from Heilongjiang Province (Huma, Xunke, Tunkhe, Harbin) belong to the *S*. *keyserlingii* species [9, 12, 13, 21]. The other samples (XM1802, XM1804, XM1882, XM2107, GQ981647, GQ981647) from Heilongjiang Province (Bin, Maoershan Mountains) and Jilin Province (Fusong) belong to the *S*. *tridactyla* species [2, 9, 11, 13]. Since the two species maybe have a parapatric distribution, it is necessary to study a possible gene flow between population on edge and species boundaries [2]. No studies, however, have ever been done to solve this problem in China, the taxonomic status of *S*. *tridactyla* in Northeast China needs to be further studied.

In this study, mitochondrial COI gene was used to study the phylogeny and more detailed information of *Salamandrella* species in Northeastern China. Because COI gene as the universal and standard DNA barcoding marker is better than other molecular markers in Hynobiids [9, 13]. We conducted a phylogenetic analysis. The analysis distinguished species in unidentified samples, delimited distribution ranges, analyzed genetic variation, assessed geographical barriers, and discussed phylogenetic relationships of the two species of *Salamandrella* more precisely. A major goal is to provide a scientific basis for formulating forward-looking strategies for cryptic species conservation in China.

## Materials and methods

### Specimens collection

Since the dispersal of *Salamandrella* genus depends on water, samples in the same basin and geographically distributed in the same village were used as a sampling point. We detected 21, 14 and 30 individuals in Greater Khingan Ridge (HM, Huma, Heilongjiang Province), Lesser Khingan Ridge (TL, Tieli, Heilongjiang Province), and four populations from Changbai Mountain (SZ, Shangzhi-zhuziying, Heilongjiang Province: 10; SC, Shangzhi-cuijia, Heilongjiang Province: 8; HL, Hailin, Heilongjiang Province: 3; and BS, Baishan, Jilin Province: 9) (see in S1 Fig and S1 Table). Material was collected during the springs and summers of 2018–2019. Specimens (cryopreservation or 95% ethanol-preserved) used are stored at the College of Life Science and Technology, Harbin Normal University (HNU), China.

### DNA extraction, PCR amplification and sequencing

Genomic DNA of frozen or 95% ethanol-preserved muscular tissue samples was extracted using the SanPrEP column animal genomic DNA extraction kit of Sangon Biotech (Shanghai) Co., Ltd. The complete sequences of the mitochondrial COI gene were amplified via polymerase chain reaction (PCR) using two pair of primers. Primer 1 (Forward: 5’-AATACACTACGAGGCTTGAT-3’; Reverse: 5’-GTAAGTGACAGAGTGGTTATG-3’; Annealing Temperature: 50°C) and Primer 2 (Forward: 5’-CTTCATGAAAGGGGCTCTACAACCCTTCATGTGGTT-3’; Reverse: 5’-AACTTGAAATTAACCTATGTGGGT-3’; Annealing Temperature: 55°C). This study utilized a newly designed primer set for COI of Hynobiid salamanders. DNA was bi-directional sequenced in the ABI 3730XL genetic analyzer by Sangon Biotech (Shanghai) Co., Ltd. Experimental protocols were approved and performed in agreement with the Institutional Animal Care and Use Committee (IACUC) of the Harbin Normal University (No.: HNUARIA2017001). All methods were carried out in accordance with relevant guidelines and regulations.

### Calculation of genetic polymorphism, distances, and differentiation

Successful sequence peak maps were analysed using Chromas 2.6.5 software.

And Blast homologous alignments (https://blast.ncbi.nlm.nih.gov/Blast.cgi) were conducted on the NCBI website to confirm the PCR product’s identity as the target gene. The sequence alignment used Clustalx 2.1 with default parameters [22]. The sequence alignment map was run with DNAMAN 9.0 and the predicted amino acid sequence was translated using Open Reading Frame Finder (ORF) (https://www.ncbi.nlm.nih.gov/orffinder). The motif distributions of the proteins were analyzed using the MEME (Motif-based sequence analysis tools-Multiple Emfor Motif Elicitation, http://meme-suite.org/tool/meme) [23, 24]. The secondary structure was predicted using the RNA fold webserver (http://rna.tbi.univie.ac.at/cgi-bin/RNAWebSuite/RNAfold.cgi) [25]. And the 3D model was predicted using SWISS-Model (http://www.swissmodel.expasy.org/) [26]. Haplotype diversity (H_d_), nucleotide polymorphism (π), average nucleotide variation (K), and variable sites (S) were calculated by using Dnasp [27]. Based on Kimura-2-Parameter (K2P), the genetic distances within and between populations were calculated by MEGA 11.0 software [28, 29]. Then, the mantel test between genetic distances and geographical distance using PC-ORD Version6 [30]. Gene flow (N_m_) was calculated by equation [N_m_ = (1/F_st_-1)/ 2] [31]. The genetic differentiation index (F_st_) between the two populations was calculated using AMOVA analysis in Arlequin 3.5 software, with 1000 repeated samples [28, 29, 32, 33]

### Phylogenetic relation and population historical dynamics

Phylogenetic trees and branches for both nucleotide and amino acid sequences were constructed using MEGA 11.0 and PhyloSuite softwares, based on the Poisson-based model (Neighbor-joining, NJ; Maximum parsimony, MP; Maximum likelihood, ML; Bayesian; Timetree). The node confidence (BP) was acquired by 1000 repeated sampling using Bootstrap [29, 34–39]. For outgroups, we used sequences of Hynobiids family that occur on Northeastern China with *S*. *keyserlingii* and *S*. *tridactyla*. Based on the Median-Joining algorithm in Network 4.6.1.1 [40], the haplotype evolutionary relationship was presented. Based on the mismatch distribution of pairwise haplotypes genetic differences in the COI gene, Tajima’s D and Fu’s Fs were used to estimate the population historical dynamics of *Salamandrella* species [41–43].

## Results

### Genetic diversity

The 65 complete sequences of mitochondrial COI gene obtained from six different geographic populations have a nucleotide length of 1550 to 1551 bp. Populations in Greater Khingan Ridge (HM) and Lesser Khingan Ridge (TL) belong to *S*. *keyserlingii*, while populations in Changbai Mountain (SZ, SC, HL, and BS) belong to *S*. *tridactyla*. Sixty-five individuals contain a total of 81 nucleotide variable sites (Table 1). Base composition analysis showed that GC content ranged from 37 to 38% (S1 Table), showing a significant AT preference and anti-G bias. This is consistent with the composition of Cyt b nucleotides in other vertebrates[44]. In the analysis of haplotype diversity (H_d_), nucleotide polymorphism (π), and average nucleotide variation (K), a total of 35 haplotypes (named *SK1–SK15* and *ST1–ST20*, under accession numbers OP050070−OP050104) were found in the COI gene. The TL population had the highest number of haplotypes (11), while the HL population had the lowest number (2). All populations showed medium-high haplotype diversity (0.607–0.978) and low nucleotide polymorphism (0.00129–0.00546) (Table 1). The motif distributions of the 35 COI proteins were analyzed utilizing the MEME. And a total of 6 conserved motifs were identified. Based on this, their secondary structure and 3D model are predicted respectively. The results suggest that all haplotypes are relatively conserved and can be divided into two branches, *S*. *keyserlingii* and *S*. *tridactyla*, based on species. Additionally, they have distinct protein structures (S2 Fig).

**Table 1:** Genetic diversity index of 6 populations of *Salamandrella* genus.

Population	Variable site	Number of haplotypes	Haplotype diversity, H_d_	Nucleotide polymorphism, π	Average nucleotide variation, K
**HM**	13	4	0.705	0.00262	4.06667
**TL**	12	11	0.956	0.00132	2.05495
**SZ**	24	9	0.978	0.00450	6.97778
**SC**	7	3	0.607	0.00224	3.46429
**HL**	3	2	0.667	0.00129	2.00000
**BS**	22	6	0.833	0.00546	8.44440
**Total**	81	35	-	-	-

Huma population is represented by HM, Tieli population is represented by TL, Shangzhi-zhuziying population is represented by SZ, Shangzhi-cuijia population is represented by SC, Hailin population is represented by HL, and Baishan population is represented by BS.

### Genetic distances and differentiation

Using DANMAN to describe the genetic distances of populations, it was discovered that these distances subdivided the six populations into two categories. HM and TL had a low genetic distance of 0.003, and therefore were clustered together. Other populations (SZ, SC, HL, and BS) cluster together (0.005–0.008). However, the genetic distance between the two classes was large (0.097–0.098). This result is almost consistent with the geographical distance of the population (r = 0.589, *P* = 0.006) (S3A Fig). The F_st_ is a measure of population differentiation due to genetic structure, and its value ranges from 0 to 1. In the present study, high genetic differentiation was observed between populations spatially isolated by a long distance. F_st_ > 0.25 in all populations, indicating greater genetic differentiation. Except for the Nm values (1 < N_m_< 4) of SZ and SC, the Nm values of other four population were all greater than 1. This suggests that gene exchange between populations is limited and weak (S3B Fig). Population molecular variation analysis revealed that, when all populations were grouped together, the variation among COI populations accounted for the majority (F_st_ = 0.94974, *P* < 0.01). However, the AMOVA analysis of COI genes in geographically close populations showed that most of the variations were found within populations (TL & HM: F_st_ = 0.32707, *P* < 0.01; SZ, SC, HL & BS: F_st_ = 0.44236, *P* < 0.01) (Table 2).

**Table 2:** Analysis of molecular variance of *Salamandrella* genus (AMOVA).

Populations	Source of variation	Degrees of freedom, df	Sum of squares	Variance component	Percentage of variation (%)	Genetic differentiation index
**All**	Between groups	5	2194.072	42.55629 Va	94.97	F_st_ = 0.94974**
	Within the group	59	132.882	2.25224 Vb	5.03	
	Total	64	2326.954	44.80853	100	-
**TL & HM**	Between groups	1	15.005	0.79570 Va	32.71	F_st_ = 0.32707**
	Within the group	33	54.024	1.63709 Vb	67.29	
	Total	34	69.029	2.43278	100	-
**SZ, SC, HL & BS**	Between groups	3	57.931	2.28838 Va	44.24	F_st_ = 0.44236**
	Within the group	26	75.003	2.88472 Vb	55.76	
	Total	29	132.933	5.17310	100	-

"*" means significant difference (*P* < 0.05).

"**" means extremely significant difference (*P* < 0.01).

### Phylogenetic relation and population historical dynamics

**Fig 1:**
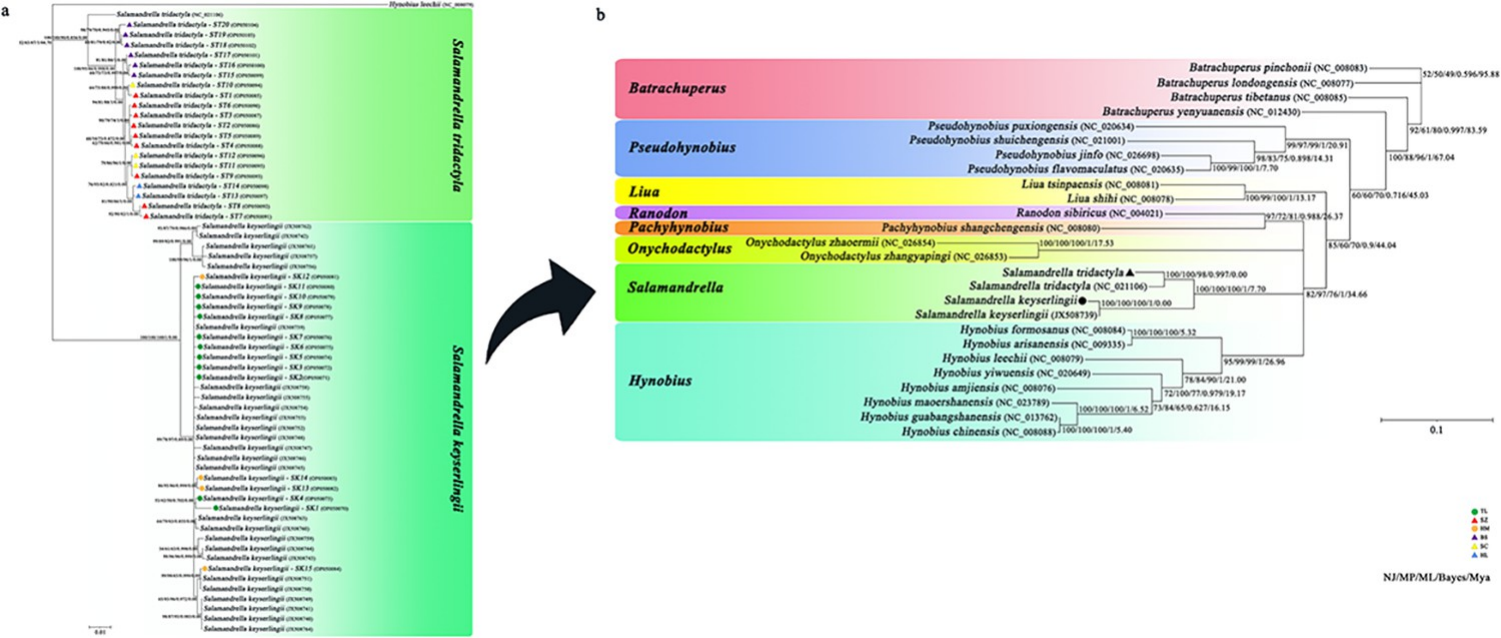
Bayesian phylogenetic tree of *Salamandrella* genus (a) and Hynobiidae family (b) based on GenBank mitochondrial COI gene. The values on the branch are bootstrap of NJ tree, bootstrap of MP tree, bootstrap of ML tree, bootstrap of Bayes tree, and time (Mya) of species differentiation from left to right. Bootstrap values from 1000 iterations were indicated above the branches.

**Fig 2:**
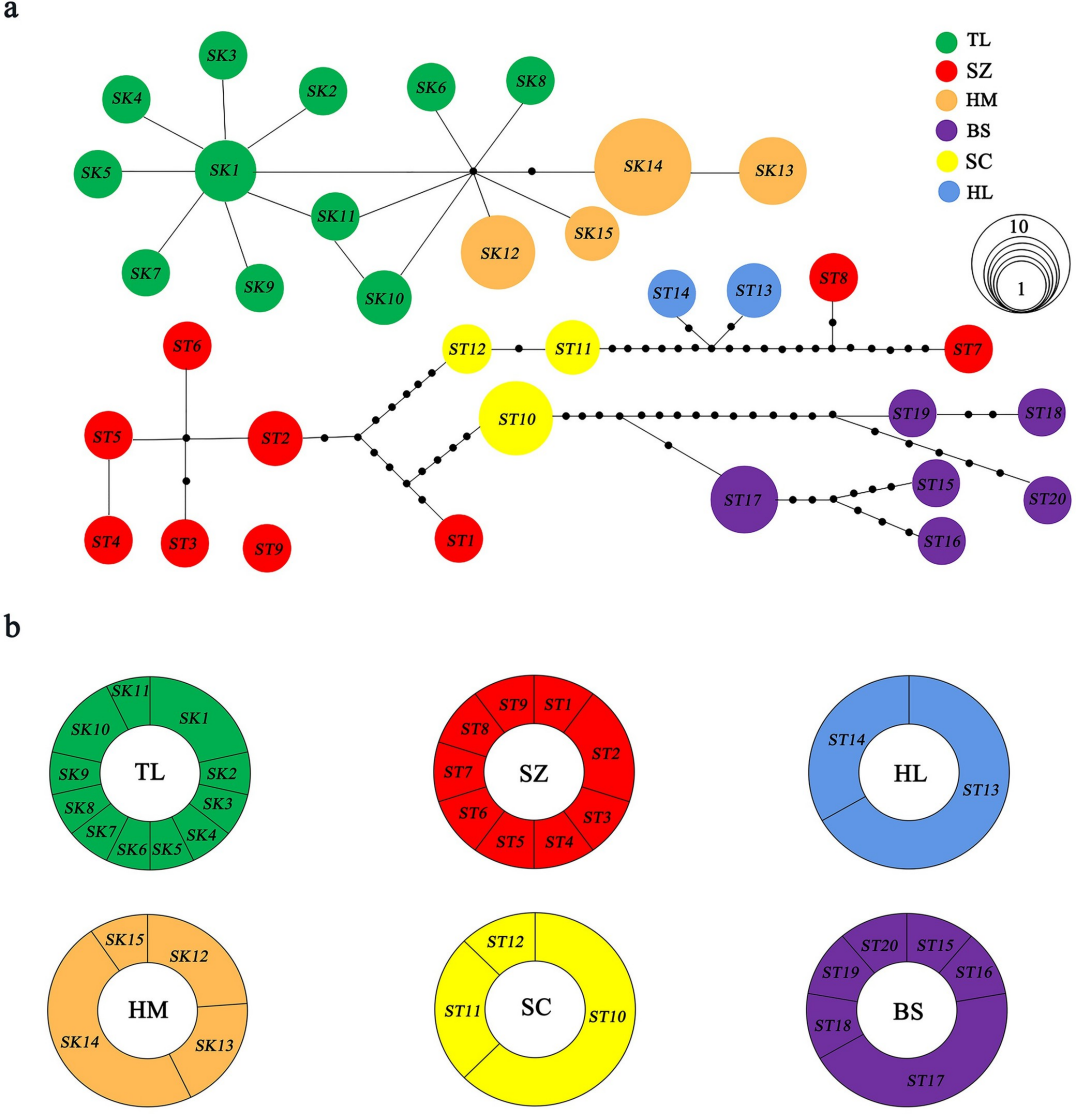
The median-joining haplotype network and haplotype distribution obtained from sampled populations of *Salamandrella* genus based on the COI gene.

The results of the phylogenetic analysis indicated a high degree of similarity between the Bayesian tree of haplotypes and the NJ, MP, ML tree. Additionally, the monophyly of the genus was strongly supported (100%) (Fig 1). All samples of *Salamandrella* species are categorized into two lineages, in line with the mountain distribution in northeast China. The findings indicate that the haplotypes of *S*. *keyserlingii* populations (HM and TL) in Khingan Range constituted a different clade from haplotypes collected from *S*. *tridactyla* populations (SZ, SC, HL and BS) in Changbai Mountains (Fig 1). Haplotype relationships were represented in a median-joining network for COI genes, which separated into two segments. Specifically, two populations from the Khingan Range (HM, TL) were linked together, while the remaining four groups from the Changbai Mountains (SZ, SC, HL, and BS) were linked together (Fig 2). Each mountain population had its own unique branch, with no shared haplotypes between populations from different mountains (Fig 2). The phylogenetic tree analysis is further supported by the haplotype network. The haplotype network was used to visualize the distribution of haplotypes in different populations. Circles represent haplotypes and the size of the ring represents the frequency of haplotypes. Similarly, it shows differences in the proportion of haplotypes in different populations (Fig 2). In the present study, using COI genes, the oldest haplotype is not found. And there was no sharing of haplotypes between the two lineages. The haplotypes are interlinked by one or more step mutations. The median vectors (Unmarked dot; Fig 2) present in the network may represent extinct or unsampled sequences in the population. By analysing both datasets, a typical star-shaped haplotype network was observed, indicating a recent population expansion following a bottleneck event [45].

The Neutral test (Table 3) and the mismatch distribution analysis (S4 Fig) of the six populations. The results showed that the values of Fu’s Fs and Tajima’s D for the TL population were negative, and the statistical results were significant (*P* < 0.05). The mismatch distribution pattern was unimodal, indicating a population expansion during the Last glacial period (between 64,000 and 210,000 years ago) for the TL population. The nucleotide mismatch distribution of the other 5 populations showed multi-modal distribution. However, both Tajima’s D and Fu’s Fs tests were not significant (all *P* > 0.05), and no expansion of population was detected (S4 Fig).

**Table 3:** Statistics of neutrality test of mtDNA COI gene of *Salamandrella* genus.

Population	HM	TL	SZ	SC	HL	BS
**Tajima’s D**	0.44945	-1.81371*	-0.96976	1.34767	0	0.13898
**Fu’s Fs statistic**	4.67867	-8.59846***	-2.78149	2.92379	1.60944	1.03047

"*" means significant difference (*P* < 0.05).

"***" means extremely significant difference (*P* < 0.001).

## Discussion

### Genetic diversity and differentiation

Population genetic diversity is a crucial component of biodiversity as it provides insights into species origin, evolution, and their ability to adapt to different environments. It also helps in understanding the level of genetic diversity, the mechanisms of its formation, and its distribution patterns. Genetic diversity is the guarantee of evolutionary potential and the basis for the conservation work. Important indices such as haplotype diversity (H_d_), nucleotide polymorphism (π), and average nucleotide variation (K) are commonly used to measure population variation [46]. In this study, we observed significant geographical differences in genetic diversity among different *Salamandrella* populations. The haplotype diversity of the six geographic groups was found to be medium-high (all H_d_ > 0.6), while the nucleotide diversity was low (all π < 0.006). This phenomenon is often observed in aquatic animals and is attributed to the expansion of a small effective population following a period of stability. It can cause lower levels of genetic diversity [47–50]. Rapid population expansion enhances the ability to maintain new mutations but results in low nucleotide diversity [51]. The low nucleotide diversity in *Salamandrella* species may be linked to geological and climate changes in the Khingan Range and Changbai Mountains, resulting in a "Bottleneck effect" and subsequent "Founder effect" in the species. This effect led to the reduction of group size and loss of genotypes, with genotype legacy only increasing after group size. Additionally, long-term geographical isolation, late sexual maturation, and limited mobility may also contribute to the extremely low level of genetic diversity [52, 53].

Studies have shown that geographical isolation or different habitats within the same water area can restrict gene exchange, leading to population differentiation in aquatic animals [54]. The coefficient of genetic differentiation (F_st_) is an important index for assessing the genetic structure of populations [55]. Large F_st_ (all > 0.25) and small N_m_ (all < 1.1) between populations indicate significant genetic differentiation. And the F_st_ (0.94974) between populations significantly affected by geographical isolation indicates that there is a high degree of genetic differentiation among *Salamandrella* species populations in Khingan Range (HM and TL populations) and Changbai Mountains (SZ, SC, HL and BS populations). It is consistent with the results of phylogenetic relationships (S3B Fig). Molecular variation analysis using AMOVA demonstrated that 94.97% of the total variation was inter-population, with the majority of variation (between 67.29%, 55.76%) being within populations from the same mountain range. This finding is consistent with the results of the haplotype phylogenetic tree (S2 Fig), population genetic distance (S3 Fig), and haplotype evolutionary network (Fig 2). These findings collectively indicate that *Salamandrella* species does not exhibit significant population differentiation within the same mountain range. It may be due to adjacent gene exchange between geographic populations that suppresses the genetic drift caused by the genetic differentiation [56], which is related to the river system connected by the Mountains.

### Phylogenetic relation and population historical dynamics

The ancestors of *Salamandrella* species separated from other Hynobiids between the Oligocene and mid-Miocene and spread around. However, interspecies competition, climatic conditions, and geographical isolation prevented the expansion of the cold-adapted ancestor of *Salamandrella* species [1, 4]. The divergence of *Salamandrella* species occurred from the end of the Tertiary Period to the early Pliocene epoch (6–8 Mya, ~7.70 Mya) [1]. Molecular data have shown that the Schrenck salamander (*S*. *keyserlingii*) is much more diverse and older than Siberian salamander (*S*. *tridactyla*) (2.5 and 0.49 Myr, respectively). The phylogenetic branch of the Schrenck newt in Northeastern China represents the earliest divergence [11]. Compared with *S*. *keyserlingii*, *S*. *tridactyla* prefers warmer climate conditions, closer to the ocean, and a smaller inland stretch to the west. When *S*. *keyserlingii* invades to the Changbai Mountains, it already exists in the region of *S*. *tridactyla* block. The population history was inferred using neutral tests and mismatch distributions. Negative values and statistically significant criteria for Fu’s Fs and Tajima’s D suggest that the sequences exhibit more nucleotide site changes than expected under the neutral evolution model, indicating a history of population expansion [42, 57]. The unimodal shape of the mismatch distribution curve, following a Poisson distribution, indicates the presence of a bottleneck effect or population expansion [58]. The neutral detection results showed that the values of Fu’s Fs and Tajima’s D of TL population were negative and deviated significantly from the neutral test (Table 3). Meanwhile, the distribution pattern showed obvious unimodal (S4 Fig). It indicated that the TL population had expansion of history during the Last glacial period, which is consistent with the results obtained by genetic diversity.

Geographical isolation and strong seismic activity may have impeded expansion and influenced species distribution. Studies confirm that amphibian migration ability is limited, and they are often affected by mountains and rivers barrier. Changes in landform and climate have affected the genetic structure of *Hyla* species [59–61]. The other example is that *Heterixalus madagascariensis* and *H*. *alboguttatus* exhibit genetic discontinuity due to Mangoro River [62]. In northeast China, the Yilan–Yitong fault zone extends from Liaodong Bay in the South, through Shangzhi and Yilan of the Heilongjiang Province, and finally reaches Luobei. The late Quaternary period saw significant tectonic activity in this area, which is the primary reason for population differentiation in species such as *Bombina orientalis* [59, 63, 64]. Our study identified two species, *S*. *keyserlingii* and *S*. *tridactyla*, with the Yilan–Yitong fault zone and Songhua River acting as geographical barriers (S1 Fig). Previous research has confirmed that *S*. *keyserlingii* mainly occupies the western side of the fault zone and Songhua River in the Khingan Range [52], while *S*. *tridactyla* is distributed in the Changbai Mountain region on the eastern side of the fault zone and Songhua River [2, 9, 11, 13]. This finding is consistent not only with our study but also with observations in Primorye Territory, Russia. However, Maoer Mountain, located at the intersection of the fault zone and Songhua River, is a unique location where both *Salamandrella* species coexist. Moreover, the distance separating *S*. *keyserlingii* (Harbin) from *S*. *tridactyla* (Bin) is less than 50 kilometers. Based on sequence variation of nuclear genome genes, we expect to find interspecific hybridization in this sympatric area similar to Russian populations (Jewish autonomous oblast) [7]. The area contains one of the range borders between the two *Salamandrella* species. However, through the analysis of various phylogenetic trees, the two species in this study belong to two branches and are completely separate. Therefore, *S*. *tridactyla* complex was not found in this study. Nevertheless, the high coefficient of differentiation of various groups within the same species may imply the existence of other possible scenarios (e.g., species complex, cryptic species, etc.). So, the Songnen Plain and Sanjiang Plain in Northeast China are the key areas worthy of study. Furthermore, *Salamandrella* species have also been recorded in the Ussuri River, Wanda Mountains and Khanka Lake. However, their specific conditions require further confirmation. Finally, the sample size in this study is inconsistent due to population size and so on, may be further studied by new techniques such as eDNA [65].

### Resources and conservation

Many species of Chinese amphibians are facing a survival crisis due to habitat loss, pollution, invasive species, and overutilization. Some of them have been listed as internationally and/or nationally protected species [66]. In China, certain species of tailed amphibians have been listed in the CITES appendix. *Salamandrella* species are endangered due to habitat disturbance caused by human activities and decreased habitat quality. Furthermore, the lack of diversity within each geographic group of *Salamandrella* species and the impact of natural environmental changes and the construction of water projects may contribute to a decline in genetic diversity and a reduced ability to adapt to complex environments. Thus this further threatens the survival of *Salamandrella* species [67, 68]. This is the main reason why *S*. *keyserlingii* was listed as a Category II National Key Protected Wild Animal in China in 2021. The low nucleotide diversity observed in this study underscores the urgent need for the preservation of its genetic diversity [69]. Conversely, *S*. *tridactyla* has not yet been listed due to insufficient evaluation of its taxonomic status in China. The results of population genetic structure and geographical pattern of pedigree system in this study can provide a scientific basis for the development of *Salamandrella* species conservation strategies [17, 59, 62, 70]. Therefore, it is of great significance to the discovery and protection of the cryptic species.

Currently, effective management and conservation measures for *Salamandrella* species have been implemented in reserves such as Khanka Lake Nature Reserve on the China-Russian border and Changbai Mountain Nature Reserve on the China-Korean border. According to the results of this study, *S*. *tridactyla* has been researched about reproductive ecology, population reintroduction, spawning habitats in these area [71–73]. However, neither this study nor previous studies on the Siberian and Primorye-Territory populations have found intermediate haplotypes of the two *Salamandrella* species [2]. The assessment of cryptic species in China has not been thoroughly evaluated, particularly in biodiversity evaluations. Furthermore, the mechanisms of morphological conservatism in cryptic species requires further investigation [74].

## Conclusion

Sequencing and analyzing the COI showed that the existence of *Salamandrella keyserlingii* (located in the Greater Khingan Ridge and Lesser Khingan Ridge) and *Salamandrella tridactyla* (located in Changbai Mountain) in northeastern China. High genetic differentiation among the species may be linked to the Yilan–Yitong fault zone and Songhua River. However, we need further study about the intermediate haplotypes and the presence or absence of sympatry between the two types.

## Supporting information

S1 Table Species information of *Salamandrella* genus in this study. (DOCX)

S1 Fig Sample map of *Salamandrella* genus. (TIF)

S2 Fig The conserved sequence of COI. (a) Characteristic features of the COI based on a phylogenetic tree and COI genes show conserved features, including (b) conserved motifs present in the amino acids. (c) The Secondary Structure of COI based on free energy minimization. (d) The 3D of COI protein.
(TIF)

S3 Fig Genetic distances, geographical distances (a), Fst, and Nm (b) among different geographic populations of Salamandrella genus. Huma population is represented by HM, Tieli population is represented by TL, Shangzhi-zhuziying population is represented by SZ, Shangzhi-cuijia population is represented by SC, Hailin population is represented by HL, and Baishan population is represented by BS. The green part in the lower left corner are the genetic distances, the blue part in the upper right corner are the geographic distances. The yellow part in the lower left corner are Fst, the orange part in the upper right corner are Nm.
(TIF)

S4 Fig The observed (solid line) and expected under the sudden expansion model (dashed line) mismatch distributions showing the frequencies of pair-wise differences of COI gene in six populations of *Salamandrella* genus. (TIF)

S1 Data Raw file of sequencing results. (ZIP)
