# Supplementary material for: Phylogenetic relationships and genetic differentiation of two Salamandrella species as revealed via COI gene from Northeastern China
Source: PLoS One. 2024 Feb 14;19(2):e0298221. doi: 10.1371/journal.pone.0298221 (PMC10866476; doi:10.1371/journal.pone.0298221)
Supplement: S1 Table — (DOCX) [file pone.0298221.s001.docx]

**Table S1**

Species information of *Salamandrella* genus in this study.

| **Species** | **Locality (Population)** | **Position** | **Number of samples** | **Haplotypes** | **GenBank accession number** |
| --- | --- | --- | --- | --- | --- |
| ***Salamandrella*** |  |  |  |  |  |
| ***S. keyserlingii*** | Lesser Khingan Ridge, Tieli, China (TL) | N46º58'19.00"/E128º42'17.21" | 3 | SK 1 | OP050070 |
| ***S. keyserlingii*** | Lesser Khingan Ridge, Tieli, China (TL) | N46º58'19.00"/E128º42'17.21" | 1 | SK 2 | OP050071 |
| ***S. keyserlingii*** | Lesser Khingan Ridge, Tieli, China (TL) | N46º58'19.00"/E128º42'17.21" | 1 | SK 3 | OP050072 |
| ***S. keyserlingii*** | Lesser Khingan Ridge, Tieli, China (TL) | N46º58'19.00"/E128º42'17.21" | 1 | SK 4 | OP050073 |
| ***S. keyserlingii*** | Lesser Khingan Ridge, Tieli, China (TL) | N46º58'19.00"/E128º42'17.21" | 1 | SK 5 | OP050074 |
| ***S. keyserlingii*** | Lesser Khingan Ridge, Tieli, China (TL) | N46º58'19.00"/E128º42'17.21" | 1 | SK 6 | OP050075 |
| ***S. keyserlingii*** | Lesser Khingan Ridge, Tieli, China (TL) | N46º58'19.00"/E128º42'17.21" | 1 | SK 7 | OP050076 |
| ***S. keyserlingii*** | Lesser Khingan Ridge, Tieli, China (TL) | N46º58'19.00"/E128º42'17.21" | 1 | SK 8 | OP050077 |
| 1. ***keyserlingii*** | Lesser Khingan Ridge, Tieli, China (TL) | N46º58'19.00"/E128º42'17.21" | 1 | SK 9 | OP050078 |
| ***S. keyserlingii*** | Lesser Khingan Ridge, Tieli, China (TL) | N46º58'19.00"/E128º42'17.21" | 2 | SK 10 | OP050079 |
| ***S. keyserlingii*** | Lesser Khingan Ridge, Tieli, China (TL) | N46º58'19.00"/E128º42'17.21" | 1 | SK 11 | OP050080 |
| ***S. keyserlingii*** | Greater Khingan Ridge, Huma, China (HM) | N51º14'44.62"/E126º40'5.59" | 5 | SK 12 | OP050081 |
| ***S. keyserlingii*** | Greater Khingan Ridge, Huma, China (HM) | N51º14'44.62"/E126º40'5.59" | 4 | SK 13 | OP050082 |
| ***S. keyserlingii*** | Greater Khingan Ridge, Huma, China (HM) | N51º14'44.62"/E126º40'5.59" | 10 | SK 14 | OP050083 |
| ***S. keyserlingii*** | Greater Khingan Ridge, Huma, China (HM) | N51º14'44.62"/E126º40'5.59" | 2 | SK 15 | OP050084 |
| ***S. tridactyla*** | Changbai Mountain, Shangzhi-zhuziying, China (SZ) | N44º55'42.58"/E127º56'37.56" | 1 | ST 1 | OP050085 |
| ***S. tridactyla*** | Changbai Mountain, Shangzhi-zhuziying, China (SZ) | N44º55'42.58"/E127º56'37.56" | 2 | ST 2 | OP050086 |
| ***S. tridactyla*** | Changbai Mountain, Shangzhi-zhuziying, China (SZ) | N44º55'42.58"/E127º56'37.56" | 1 | ST 3 | OP050087 |
| ***S. tridactyla*** | Changbai Mountain, Shangzhi-zhuziying, China (SZ) | N44º55'42.58"/E127º56'37.56" | 1 | ST 4 | OP050088 |
| ***S. tridactyla*** | Changbai Mountain, Shangzhi-zhuziying, China (SZ) | N44º55'42.58"/E127º56'37.56" | 1 | ST 5 | OP050089 |
| ***S. tridactyla*** | Changbai Mountain, Shangzhi-zhuziying, China (SZ) | N44º55'42.58"/E127º56'37.56" | 1 | ST 6 | OP050090 |
| ***S. tridactyla*** | Changbai Mountain, Shangzhi-zhuziying, China (SZ) | N44º55'42.58"/E127º56'37.56" | 1 | ST 7 | OP050091 |
| ***S. tridactyla*** | Changbai Mountain, Shangzhi-zhuziying, China (SZ) | N44º55'42.58"/E127º56'37.56" | 1 | ST 8 | OP050092 |
| ***S. tridactyla*** | Changbai Mountain, Shangzhi-zhuziying, China (SZ) | N44º55'42.58"/E127º56'37.56" | 1 | ST 9 | OP050093 |
| ***S. tridactyla*** | Changbai Mountain, Shangzhi-cuijia, China (SC) | N44º55'42.87"/E127º56'39.99" | 5 | ST 10 | OP050094 |
| ***S. tridactyla*** | Changbai Mountain, Shangzhi-cuijia, China (SC) | N44º55'42.87"/E127º56'39.99" | 2 | ST 11 | OP050095 |
| ***S. tridactyla*** | Changbai Mountain, Shangzhi-cuijia, China (SC) | N44º55'42.87"/E127º56'39.99" | 1 | ST 12 | OP050096 |
| ***S. tridactyla*** | Changbai Mountain, Hailin, China (HL) | N44°43'48.07"/E129°12'15.29" | 2 | ST 13 | OP050097 |
| ***S. tridactyla*** | Changbai Mountain, Hailin, China (HL) | N44°43'48.07"/E129°12'15.29" | 1 | ST 14 | OP050098 |
| ***S. tridactyla*** | Changbai Mountain, Baishan, China (BS) | N42º35'52.90"/E127º50'30.94" | 1 | ST 15 | OP050099 |
| ***S. tridactyla*** | Changbai Mountain, Baishan, China (BS) | N42º35'52.90"/E127º50'30.94" | 1 | ST 16 | OP050100 |
| ***S. tridactyla*** | Changbai Mountain, Baishan, China (BS) | N42º35'52.90"/E127º50'30.94" | 4 | ST 17 | OP050101 |
| ***S. tridactyla*** | Changbai Mountain, Baishan, China (BS) | N42º35'52.90"/E127º50'30.94" | 1 | ST 18 | OP050102 |
| ***S. tridactyla*** | Changbai Mountain, Baishan, China (BS) | N42º35'52.90"/E127º50'30.94" | 1 | ST 19 | OP050103 |
| ***S. tridactyla*** | Changbai Mountain, Baishan, China (BS) | N42º35'52.90"/E127º50'30.94" | 1 | ST 20 | OP050104 |
