# Supplementary figures and images for: Phylogenetic relationships and genetic differentiation of two Salamandrella species as revealed via COI gene from Northeastern China
Source: PLoS One. 2024 Feb 14;19(2):e0298221. doi: 10.1371/journal.pone.0298221 (PMC10866476; doi:10.1371/journal.pone.0298221)

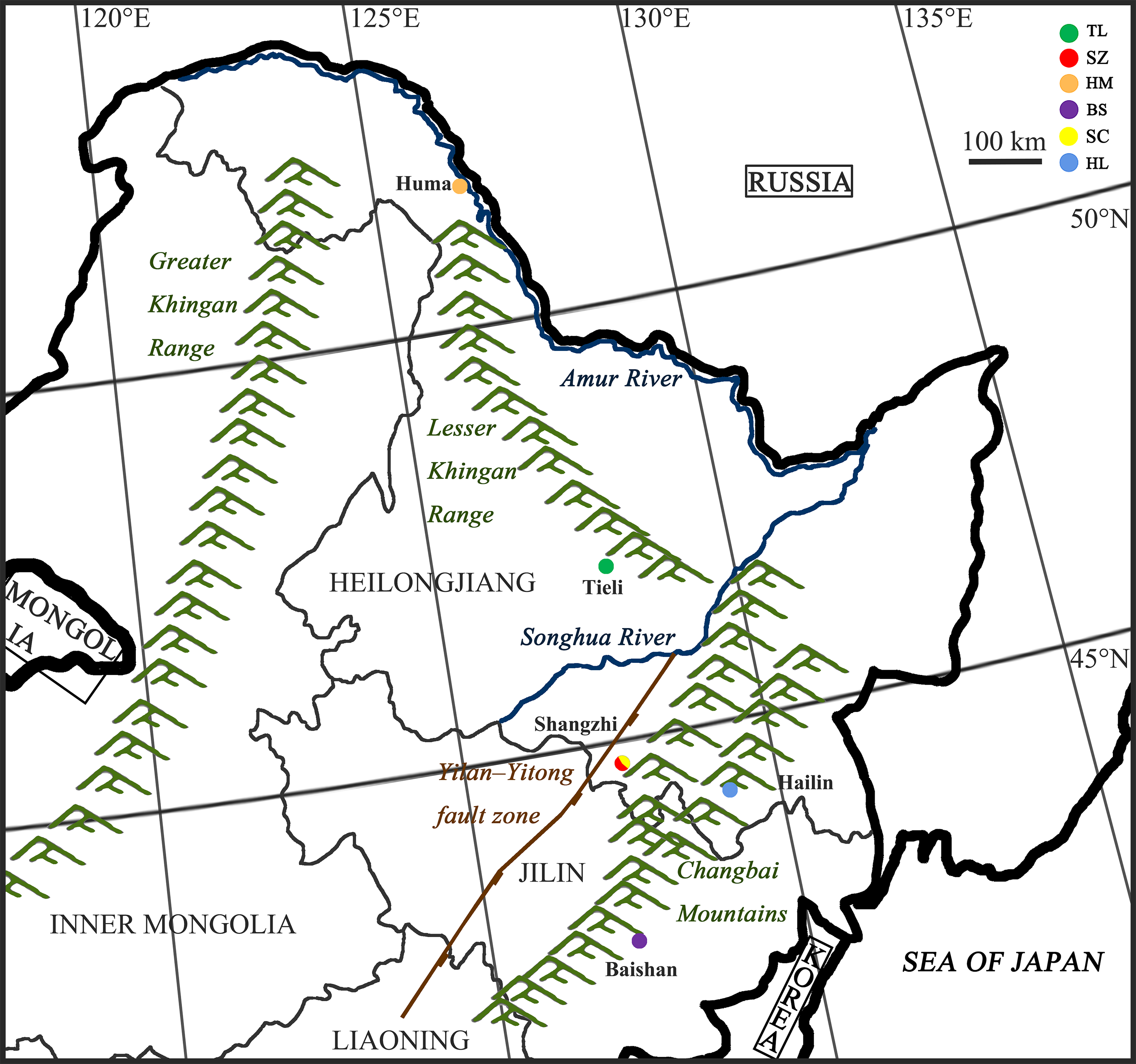

Supplement: S1 Fig — (TIF) [file pone.0298221.s002.tif]

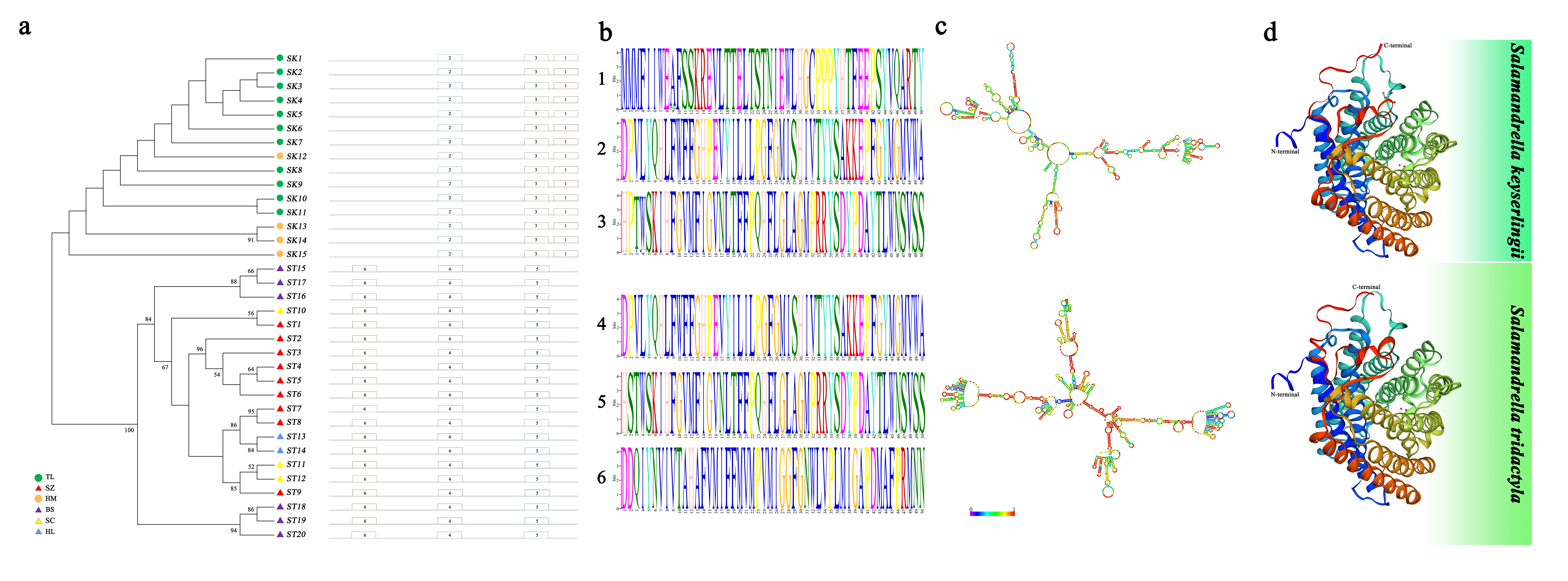

Supplement: S2 Fig — (a) Characteristic features of the COI based on a phylogenetic tree and COI genes show conserved features, including (b) conserved motifs present in the amino acids. (c) The Secondary Structure of COI based on free energy minimization. (d) The 3D of COI protein. (TIF) [file pone.0298221.s003.tif]

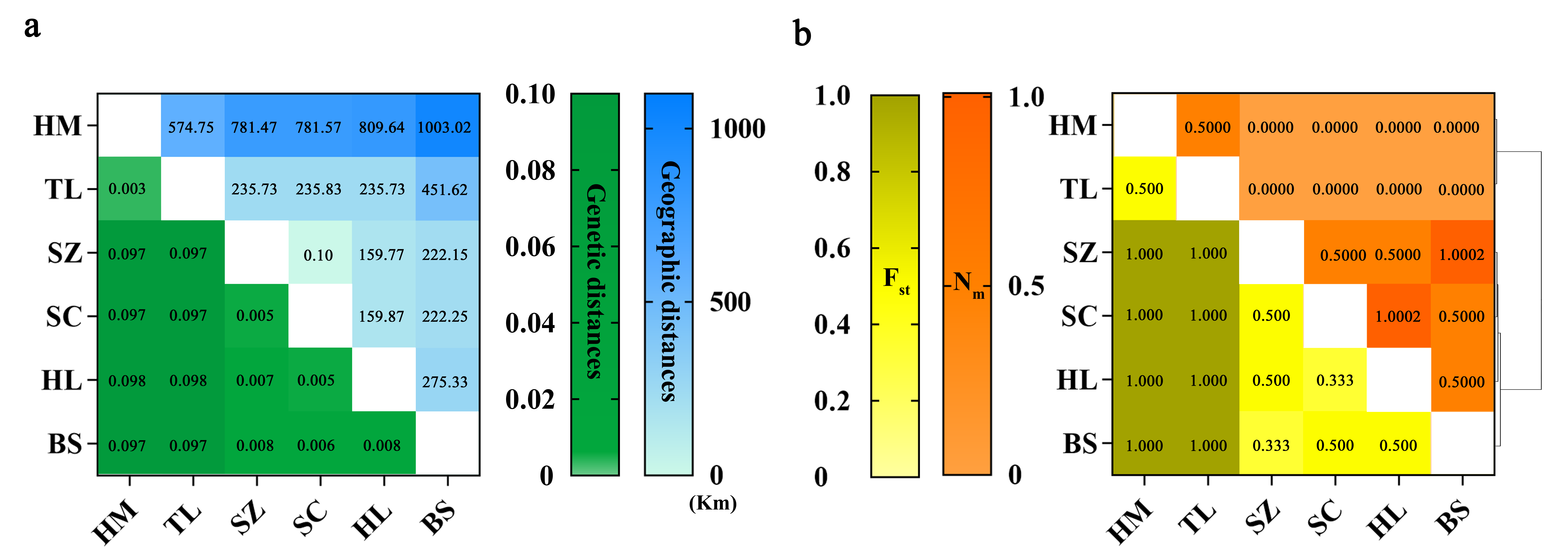

Supplement: S3 Fig — Genetic distances, geographical distances (a), Fst, and Nm (b) among different geographic populations of Salamandrella genus. Huma population is represented by HM, Tieli population is represented by TL, Shangzhi-zhuziying population is represented by SZ, Shangzhi-cuijia population is represented by SC, Hailin population is represented by HL, and Baishan population is represented by BS. The green part in the lower left corner are the genetic distances, the blue part in the upper right corner are the geographic distances. The yellow part in the lower left corner are Fst, the orange part in the upper right corner are Nm. (TIF) [file pone.0298221.s004.tif]

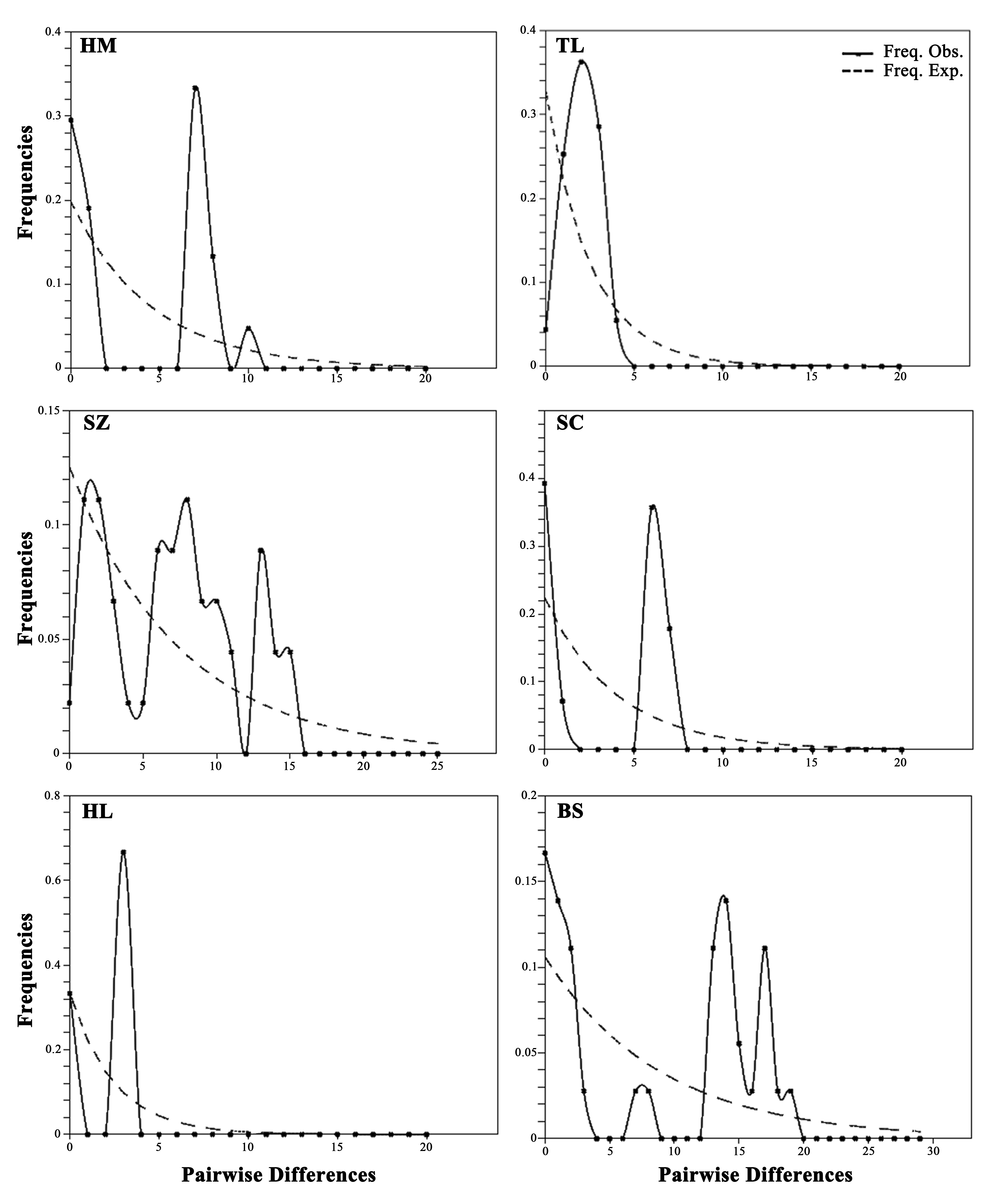

Supplement: S4 Fig — (TIF) [file pone.0298221.s005.tif]
